# Learning from pandemic responses: Informing a resilient and equitable health system recovery in Thailand

**DOI:** 10.3389/fpubh.2023.1065883

**Published:** 2023-01-25

**Authors:** Viroj Tangcharoensathien, Jos Vandelaer, Richard Brown, Rapeepong Suphanchaimat, Phiangjai Boonsuk, Walaiporn Patcharanarumol

**Affiliations:** ^1^International Health Policy Programme, Ministry of Public Health, Nonthaburi, Thailand; ^2^WHO Country Office, Nonthaburi, Thailand

**Keywords:** COVID-19, pandemic response, equitable health system recovery, resilient, adaptive strategies, Thailand

## Abstract

This article is part of the Research Topic ‘Health Systems Recovery in the Context of COVID-19 and Protracted Conflict’. The third quarter of 2022 saw COVID-19 cases and deaths in Thailand reduced significantly, and high levels of COVID-19 vaccine coverage. COVID-19 was declared an “endemic” disease, and economic activities resumed. This paper reviews pre-pandemic health systems capacity and identifies pandemic response strengths, weaknesses and lessons that guided resilient and equitable health system recovery. Robust health systems and adaptive strategies drive an effective pandemic response. To support health system recovery Thailand should (1) minimize vulnerability and extend universal health coverage to include migrant workers and dependents; (2) sustain provincial primary healthcare (PHC) capacity and strengthen PHC in greater Bangkok; (3) leverage information technology for telemedicine and teleconsultation; (4) enhance and extend case and event-based surveillance of notifiable diseases, and for public health threats, including pathogens with pandemic potential in wildlife and domesticated animals. This requires policy and financial commitment across successive governments, adequate numbers of committed and competent health workforce at all levels supported by over a million village health volunteers, strong social capital and community resilience. A strengthened global health architecture and international collaboration also have critical roles in establishing local capacities to develop and manufacture pandemic response products through transfer of technology and know-how. Countries should engage in the ongoing Inter-government Negotiating Body to ensure a legally binding instrument to safeguard the world from catastrophic impacts of future pandemics.

## 1. Introduction

As of 25 September 2022, Thailand reported 4.7 million COVID-19 cases, and 32,721 deaths; equivalent to 65,329 cases and 456.8 deaths per million population (1). Thailand ranks 142^nd^ and 137^th^ globally in terms of cases and deaths per million population. COVID-19 vaccine rollout began in May 2021; by September 2022, 79.6% of the Thai population were fully vaccinated and 44.7% had received booster doses (2).

Wilasang et al. (3) estimated excess deaths in 2021 at 14.3% (95%CI: 8.6–18.8%) higher than the expected mortality projected from the last five years. Another study estimated excess deaths between 2020 and 2021 at 24.9 per 100,000 population, compared with reported deaths of 15.3 per 100,000 population (4). This rate is considerably lower than the global all-age rate of 120.3.

In 2021, Thailand ranked fifth out of 195 countries and territories for the Global Health Security Index (GHSI), with an index score of 68.5 after US, Australia, Finland and Canada. Though the six domains of GHSI, namely prevention capacity, detection and reporting, rapid response, health system capacity, compliance with international norms and risk environment are useful for analysis of pandemic preparedness and response capacity, higher GHSI scores do not consistently predict better control outcome. For example, a study has shown a positive association between GHSI and COVID-19 cases and deaths, but this is not related to the COVID-19 testing rate (*r* = 0.35, *P* < 0.001) (5). This counter-intuitive outcome is also confirmed by another study on discrepancies between the GHSI and the actual performance in OECD countries; probably the effect of leadership was not adequately covered by the index (6). Governance and leadership are keys for effective pandemic management (7). Further, domains often viewed as external to the health sector are central determinants of health system resilience in pandemic response including governance, finance, collaboration across sectors and community engagement (8). None of these are elements of the GHSI.

The third quarter of 2022 saw a significant reduction in the number of COVID-19 cases, and high levels of vaccine coverage in Thailand. The government declared COVID-19 an “endemic” disease, fully resumed economic activity and initiated a plan for health system recovery.

**Figure 1:**
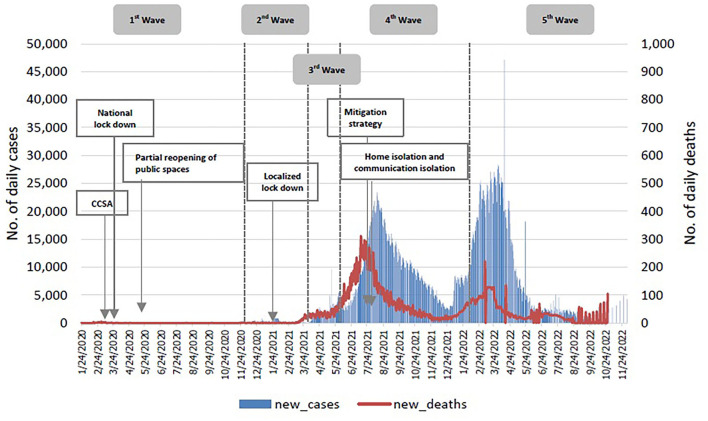
Thailand COVID-19 daily reported cases, February 2010 to September 25, 2022. Source: COVID-19 Corona Virus Pandemic (1).

Figure 1 shows COVID-19 case numbers and deaths, together with policy interventions, i.e. elimination in wave 1, suppression in wave 2 and 3 and mitigation in wave 4 (the peak of Delta strain transmission) when home and community isolation policies were introduced (9). In wave 5 (Omicron variant), the country endorsed a “living with COVID” strategy. Vaccine rollout was expedited in early 2021.

This paper is based on the experience of policy actors from Thailand's Ministry of Public Health and the WHO Country Office. In this paper, we argue that a leading reason for Thailand's success in dealing with COVID-19 was the country's robust pre-pandemic health system. This was supported by an effective pandemic response, through whole-of-government and whole-of-society approaches, and decisive decision-making informed by science, agility, and adaptivity. Response challenges included significant vulnerable populations (especially migrant workers), poverty and sub-optimal primary health care in Bangkok, and politicization of the pandemic, and particularly of the vaccine debate.

These experience-based observations were further complemented by focused Google literature searches in three areas: (1) pre-pandemic health system resilience including primary healthcare, health workforce and universal health coverage; (2) enabling factors, and (3) challenges faced during the 3-year pandemic.

## 2. Pre-pandemic: Robust health systems

A robust health system is a critical foundation for pandemic response. A study further proposes health-system integration across UHC and global health security, innovative and unified health financing, cross-sector resilience and equity as core values (10).

Thailand's health system is dominated by the public sector. In 2021, the Ministry of Public Health (MOPH) was the major healthcare provider in the country, maintaining 68% and 67% respectively of the 1,367 hospitals and 167,563 beds nation-wide, and providing for 64% and 71% of all outpatient visits and inpatient cases. Other public sector providers such as Defense, Universities and local government had a very limited healthcare provision role. The private sector had a correspondingly smaller role, with a 24% and 20% shared of hospitals and beds; and a 23% and 21% shared of total outpatient visits and inpatient cases in 2021 (11).

Robust government health systems were achieved through four decades of investment in health infrastructure until full geographical coverage of health centers, district hospitals and provincial hospitals in all sub-districts, districts and provinces, respectively was achieved. District health systems provide a comprehensive range of services including integrated public health functions, and are the foundation for UHC with favorable access outcomes (12).

Since 2002, the whole population is covered by one of three public health insurance schemes. Benefit packages are comprehensive, resulting in high financial risk protection (13); which together with geographical coverage of health services results in a low level of unmet healthcare needs (14, 15). The UHC service coverage index increased from 41% in 2000 to 83% in 2019 (16), while the proportion of the population spending more than 10% of their household consumption on out-of-pocket health care expenditure reduced from 5.63% in 2002 (prior to UHC) to 1.87% in 2019 (17).

Scaling up and diversifying training has increased the health workforce density. The number of physicians, nurses and midwives per 1,000 population increased from 0.93 in 1991 to 4.07 in 2019 against the target of 4.45 physicians, nurses and midwives per 1,000 population by 2030 (18). Since 1974, Thailand has had special tracks to recruit rural students into medical and nursing careers, later extended to dentistry and pharmacy, with the expectation that they return to work in their communities after graduation (19). Evidence suggests this initiative achieves better results in terms of fulfilling a 3-year mandatory rural service requirement, and higher clinical competencies (20).

In 1980, MOPH launched a 2-year Field Epidemiology Training Programme for medical, veterinary and other health science graduates. Joint training between human and animal health sectors has improved surveillance and control of zoonotic diseases, and improved collaboration among One Health partners (21). Further, 1-4 weeks short courses on basic epidemiology are also provided to health officers as well as a 6–12 month intermediate level course. MOPH also oversees 1,030 Surveillance and Rapid Response Teams (SRRTs) in districts, provinces and centrally.

## 3. Pandemic responses: Key enabling factors

An inter-country study demonstrated that in Thailand, cross-sectoral coordinated action, an effective test, trace, quarantine, treatment system and effective governance to ensure adherence to public health and social measures were all important factors that contributed to the national pandemic response (22).

A Joint Intra-action Review of Thailand's responses to COVID-19 by WHO and the MOPH also identified decisive leadership informed by science, agility and adaptivity, and adequate numbers of qualified and committed cadres of health professionals as enabling factors (23).

A whole-of-government approach to pandemic response was facilitated through the establishment in April 2020 of the Center for COVID-19 Situation Administration (CCSA). The CCSA was chaired by the Prime Minister, supported by various Ministry Emergency Operation Centers and led by respective permanent secretaries. The MOPH oversaw epidemiological monitoring, introduced public health and social measures and supported healthcare delivery. The Ministry of Labor dealt with unemployment and migrant workers. The Ministry of Finance mobilized budget for pandemic containment and support to affected populations. The CCSA delegated authority for COVID-19 management to provincial governors, supported by multi-sectoral provincial disease control committees.

Containment strategies ranging from elimination, suppression and mitigation were guided by the rapidly evolving situation. An initial goal in April 2020 to achieve elimination through a “nation-wide lock-down” significantly interrupted transmission, but with a corresponding negative economic impact. In response to the larger subsequent wave in December 2020, the government instead aimed at suppressing localized transmission through “targeted lock-downs,” so that the number of severe cases was kept within the total Intensive Care Unit bed capacity; while in unaffected areas, economic activities continued (9). Later evolution of the pandemic, including emergence of the Delta variant in the third quarter of 2021, led to a very large surge of daily cases and deaths, requiring the adoption of mitigation and triage strategies to prevent hospitals from becoming overwhelmed. This meant that severe cases were allocated to hospital with ICUs, while mild cases were treated at home or in the community. The moderately unwell cases received care in field hospitals, some equipped with oxygen generators and ventilators. In addition, with support from government, the private sector and communities, an adequate number of small to large-scale field hospitals (data on number of field hospitals was incomplete) were established, with basic equipment and treatment capacities.

To ensure access to care, the government approved funds to provide COVID-19 related services to all people, including the non-Thai population by purchasing services from public and private healthcare providers using the same rules, regulations and payment rates (24). Treatment and provision of food at home, in community isolation facilities and field hospitals were subsidized by the government. Budget was rapidly disbursed for frontline pandemic control while ensuring accountability and transparency of budget execution (25).

A whole-of-society approach was adopted, whereby citizens, the private sector and civil society worked together to mitigate the impact on vulnerable populations. Strong social capital was demonstrated by a voluntary “food pantry” initiative, through which individuals, communities, temples and mosques would fill and refill food and essential items into community-based “pantries” to support individuals who had been made redundant or were unemployed (26, 27). This societal fabric and the spirit of helping others reflects the generosity and hospitality seen among Thais. Frontline health workers, ICU staff and public health officers all contributed significantly during the pandemic, especially during the roll out of vaccination nation-wide (28), and their roles are fully recognized and appreciated (29).

Starting in 2020, Surveillance and Rapid Response Teams working at local level were complemented and supported by 1.04 million village health volunteers (VHVs) in communities. These volunteers are the unsung heroes of the pandemic response and continue to play a significant role in supporting surveillance (30), mitigating impact and supporting pandemic control (31). VHVs have created pluralistic “socio-political networks” with community stakeholders, local officials and private sector actors to support COVID-19 mitigation measures (32). Since 2009, each volunteer has received a monthly honorarium of 600 Thai Baht for their contribution; this was adjusted up to 1,000 Thai baht (US$ 32) in 2019. During the time of COVID, the government subsequently approved an additional monthly payment of 500 baht in recognition of their contribution. Other incentives include compensation to their families if VHVs die from COVID-19.

Teleconsultation was applied to support patients under home isolation, to provide counseling on self-care and treatment and ensure confidence for their return to the community after recovery (33). Clinical pharmacists also provided telemonitoring, counseling and pharmaceutical care for COVID-19 patients (34). Telehealth was applied to support compliance and continuation of antiretroviral therapy among people living with HIV/AIDS (35). In order to maintain essential health services, notably NCDs, face-to-face outpatient visits that could increase the risk of COVID-19 infection were replaced by telemedicine, teleconsultation and postal delivery of medicines.

## 4. Pandemic response: Challenges and failures

Some fundamental pandemic response challenges and vulnerabilities were exposed, especially in urban areas, including many unregistered migrants; the complexity of managing urban slums; a political culture of polarization and conflict; and an imbalance between public health capacity and needs in a metropolis like Bangkok. Bangkok has a significant level of autonomy and is densely populated, with pockets of extreme deprivation. While the pandemic response aimed to deal with these enormous challenges, entrenchment in bureaucracy meant that they hampered and undermined the response. It remains to be seen whether well-intentioned attempts to overcome these challenges may have triggered long overdue positive changes.

Labor trafficking results in a large proportion of unregistered migrants in Thailand, mostly from neighboring countries. A lack of coherent policy on migrant health insurance (36), tightly packed accommodation that makes physical distancing impractical (37), and challenges in access to healthcare (38), all likely played a role in these communities becoming amplifiers of outbreaks that proved difficult to control (39).

In 2018, 23.7% of Thai urban populations were living in slums (40). A survey in 2018 reported 638 slum communities in Bangkok with 0.579 million residents living in 146,462 households (41). These figures exclude an unknown number of internal Thai migrants from other provinces, and unregistered non-Thai migrants.

A study in urban slums reported that during the pandemic, a significant proportion of residents had to limit their food and nutrient consumption. Almost one-tenth of the participants relied on donated food only. The majority of them (61.1%) could not access an income compensation scheme. As a result, COVID-19 forced Bangkok slums residents to live below the subsistence level in multiple ways, with limited access to social protection (42).

Political conflict during the pandemic presented significant challenges. Four Parliamentary “Distrust Debates” were organized by opposition parties against the Prime Minister and selected Ministers. Distrust Debates can lead to resignations of distrusted Minister(s), or dissolution of the Cabinet if the Prime Minister was “distrusted.” The first distrust debate was convened on 24 to 27 February 2020, the second from 16 to 19 February 2021, the third from 31 August to 3 September 2021, and the most recent from 19 to 22 July 2022. For all these four debates, a vote in favor of distrust was defeated. Two general debates were also convened, during which vaccine-related issues were hot topics.

COVID-19 vaccination started in May 2021 (initially with limited supplies) and was significantly scaled up in the last quarter of 2021. Concerns raised by opposition parties during the distrust and general debates referred to pandemic control, socio-economic impact and vaccines. Criticism included the use of inactivated vaccines (Sinovac and Sinopharm) despite WHO Emergency Use Authorization, and issues related to immunogenicity and safety of heterogeneous vaccine schedules. Key accusations made included that Thai people were being used as guinea pigs for testing heterologous vaccine schedules (43). Accusations were addressed through the presentation of evidence but this increased the burden of MOPH communication activities, and led to both public confusion and a lack of confidence in vaccine quality and effectiveness. Dis-information and fake news about mortality from adverse events associated with vaccination further complicated the situation (44).

Evidence also emerged after these debates that further disproved opposition party claims: for example, the WHO Strategic Advisory Group of Experts on Immunization (SAGE) subsequently recommended heterologous vaccine schedules based on published evidence, including four studies by Thai scientists that were cited as SAGE references (45). Recent evidence from real-world surveillance data has also confirmed that heterologous vaccination schedules provided significant benefit in reducing cases and deaths comparable to, or even greater than some homologous vaccine schedules (46).

Pandemic responses also faced challenges in urban settings. Bangkok has a registered population of 5.5 million, as well as 2.35 million non-registered individuals and a daily-commuter population of 0.55 million (47). The Bangkok Metropolitan Administration (BMA), has a legal mandate for health, but sub-optimal public health capacity with relatively few Surveillance and Rapid Response Teams, only 69 primary health care centers and just 10,577 health volunteers. This proved insufficient for pandemic response when compounded by ineffective collaboration across government agencies and contributed to Bangkok being an epi-center of poorly controlled COVID-19 infection, and on occasions contributing to nation-wide spread of infection.

**Figure 2:**
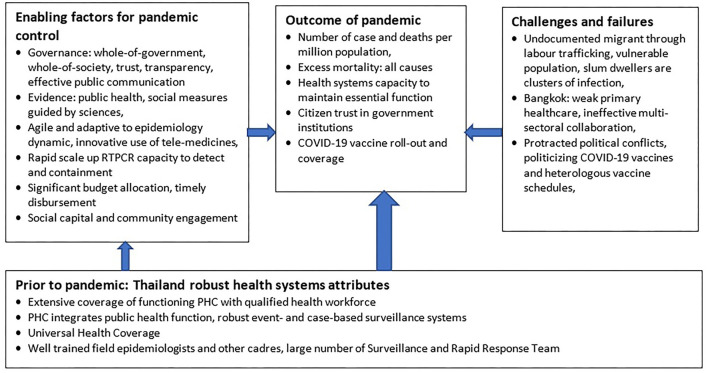
Factors contributing to pandemic outcomes.

Figure 2 summarizes the key findings. Despite political conflict and challenges to healthcare in urban settings, the pre-existing robust health system in Thailand synergized with key enabling factors led to an effective pandemic response.

## 5. Thailand's next steps in building back better, fairer and more resilient health systems

### 5.1. Strengthen capacities to generate evidence to inform policies

Three priorities for evidence generation have been identified and relevant actions taken in collaboration with the scientific community and the social welfare sector: these are long COVID, orphanhood and health threats at the human-animal interface.

A systematic review reports the most common post COVID symptoms as weakness, general malaise and fatigue; while 37% of patients reported reduced quality of life and reduced pulmonary function (48). The research community should establish prospective cohorts to assess post COVID symptoms, and mortality outcomes.

Global estimates of COVID-19 related orphanhood exist (49), but Thailand lacks data. The International Health Policy Programme, a research arm of MOPH, is working with stakeholders to directly estimate the number of parental orphans from the Civil Registration system. Support is critical because consequences can include abuse, traumatic grief, mental health problems, adolescent pregnancy and poor educational outcomes, especially in young orphans (50). Findings will inform a financial assistance policy by the Ministry of Social Development and Human Security.

Further, with support from the WHO Country Cooperation Strategy, the MOPH is developing a provincial One Health Capacity self-assessment tool (51, 52) to support identification of threats at the human-animal-environment interface.

### 5.2. Maximize use of information technology

The use of telemedicine should be maximized to reduce the need for outpatient services (notably for NCDs) and support virtual consultations with primary healthcare workers. The National Health Security Office has financed refills of medications by certified private pharmacies in the community. Mobile applications for outpatient appointments can reduce waiting times, minimize overcrowding and increase client satisfaction (53).

### 5.3. Minimize vulnerability: Universal health coverage and access

We recommend extending UHC from the Thai population to everyone including migrant workers and their dependants. The estimated economic contribution of immigrant workers was 4.3%−6.6% of Thailand's gross domestic product in 2010, while they represented 4.7% of the employed population (54). Vaccines covered by the National Immunization Programme should be available to all children regardless of nationality, as the cost of outbreak response and containment in the community is higher if they are not fully immunized (55, 56). The MOPH should ensure funding to achieve this end. Migrants also have higher prevalence of tuberculosis (57). Although detection and treatment of tuberculosis for the non-Thai population is fully subsidized, either by the government or through Migrant Health Insurance schemes, performance of tuberculosis case detection has yet to improve.

### 5.4. Strengthen urban primary healthcare

There is an urgent need to strengthen urban primary healthcare and related public health functions including detection and reporting of notifiable diseases to facilitate timely risk assessment and response actions.

The newly elected Bangkok Metropolitan governor, Dr. Chatchart Sittipunt, has committed to strengthen primary health care in Bangkok in his policy portfolio. A Civil Society Organization's white paper on comprehensive measures to strengthen health, education, welfare and safety in Bangkok was also well received (58). Closer collaboration between the National Health Security Office and the BMA Health Department in strengthening UCS budget execution is underway (59). We also recommend extending health volunteer schemes beyond congested urban communities to cover condominiums and middle-class residential areas.

These recommendations are in line with suggestions by other organizations. For example, OECD advocates for the systematic application of science to inform policies in times of COVID-19 (60). The International Consortium of Primary Care Big Data Researchers supports continued use of virtual visit modalities in the pandemic recovery phase (61). The UCL Institute of Health Equity advocates for reducing structural inequality and vulnerability not only for a future pandemic, but for a fairer, healthier society (62). The need to strengthen urban PHC has been advocated for in a variety of country settings (63, 64).

## 6. Conclusion

The framing of this paper, see Figure 2, may have missed literature that identifies pandemic control determinants, both positive and negative. However, this policy and practice review paper summarizes tacit knowledge and hands-on experience among policy actors from the MOPH and WHO through 3 years of supporting Thailand's COVID-19 response. While any set of policies and practices is likely to be incomplete, the one offered here should be considered when evaluating national COVID-19 responses, and when steps toward health systems recovery are advanced by low- and middle-income countries. The descriptions of both good practices and challenges will, hopefully, support policy and decision makers from other countries and the global community in dealing with future public health emergencies and in building back better, fairer and more resilient health systems.

Country level actions to improve preparedness for future pandemic and public health emergencies are essential but not sufficient. A robust global health architecture and meaningful international collaboration are critical both to strengthen local manufacturing capacity of pandemic response products through transfer of technology and know-how, and to address the inequitable access seen in the global COVID-19 pandemic response. All WHO member states need to actively engage in the ongoing Inter-government Negotiating Body and negotiate for a legally binding instrument to better safeguard the world from catastrophic impacts of future pandemics.

## Author contributions

All authors listed have made a substantial, direct, and intellectual contribution to the work and approved it for publication.
